# The accuracy of multiple regression models for predicting the individual risk of recurrent lateral patellar dislocation

**DOI:** 10.1186/s12891-023-07094-2

**Published:** 2023-12-06

**Authors:** Jiang Yu, Yijin Li, Kaibo Zhang, Runze Yang, Xiaolong Yang, Meng Gong, Cheng Long, Weili Fu

**Affiliations:** 1 Department of Orthopedic Surgery/Orthopedic Research Institute, West China Hospital, Sichuan University, Chengdu, China; 2 Laboratory of Clinical Proteomics and Metabolomics, Institutes for Systems Genetics, Frontiers Science Center for Disease-related Molecular Network, National Clinical Research Center for Geriatrics, West China Hospital, Sichuan University, Chengdu, China

**Keywords:** Knee, Patella, Lateral patellar dislocation, Outcome measure, Validation

## Abstract

**Background:**

Recurrent lateral patellar dislocation (RLPD) poses a significant threat to patients’ quality of life due to knee pain, patellofemoral cartilage damage, and potential traumatic arthritis. Predictive scoring systems have been developed to assess the risk of RLPD; however, their relative accuracy remains uncertain.

**Purpose:**

To investigate the accuracy of the multiple regression models to predict the individual risk of recurrent LPD.

**Methods:**

The Patellar Instability probability calculator (PIP), Recurrent Instability of the Patella Score (RIP), and Patellar Instability Severity Score (PIS) scoring rules were measured in 171 patients with a history of patellar dislocation and 171 healthy individuals. Three prediction models were calculated based on the data to predict the risk of recurrent lateral patellar dislocation. The inter-observer and intra-observer reliability of each measurement parameter was evaluated. The predictive capacity of the three-prediction model was investigated using the receiver operating characteristic curve.

**Results:**

In the case group of 171 patients, PIS accurately predicted recurrent lateral Patella dislocation in 143 patients. RIP was 96, and PIP was 83. The positive predictive values were 92.9%, 64%, and 68% respectively. In the control group of 171 patients, the PIS was validated in 160 patients who would not experience dislocations. RIP was 117, and PIP was 50. The negative predictive values were 85.1%, 60.9%, and 36.2%, respectively. The area under the curve score for the PIS was 0.866, and the RIP was 0.673. the PIP was 0.678.

**Conclusion:**

RIP and PIP did not work to predict LPD. PIS can accurately predict recurrent lateral patellar dislocation. It can aid doctors in making treatment decisions.

**Level of evidence:**

Level III, retrospective comparative study.

## Background

Lateral patellar dislocation (LPD) is a disabling condition most prevalent in younger people and more frequently seen in females than males. [1] The incidence rate of primary lateral patellar dislocation is approximately 0.23–0.42‰. Recurrent dislocation after nonsurgical treatment accounts for approximately 7.7-78.5% of cases. [2, 3] Recurrent lateral patellar dislocation (RLPD) can cause knee pain, patellofemoral cartilage damage, and traumatic arthritis, affecting the quality of life and increasing the patient’s economic burden. [4] How to identify patients with a high incidence of RLPD early and adopt the best intervention measures is a problem that doctors want to solve. In 2014, Balcarek et al. proposed a multiple regression model called the patellar instability severity (PIS) score, which includes six variables to determine high-risk patients for LPD recurrence. [5] Because of its strong practicality and accuracy, it has become a typical application of multiple regression models. Hevesi et al. based on an extensive geographic database containing over 500,000 patients and advanced a statistical model for predicting long-term recurrence risk after first-time dislocation called the recurrent instability of the patella score (RIP) in 2019. [6] RIP holds significant potential clinical utility for determining patients at high risk for recurrent instability after a primary patellar dislocation. In 2022, Wierer et al. recently established multiple regression models to Predict the Individual Risk of RLPD named The Patellar Instability Probability Calculator (PIP). [7] PIP is proposed to estimate the individual risk of early recurrence when counseling patients after primary LPD. The establishment of the above three predictive scoring systems was based on multiple regression models, and the predictive indicators included were somewhat different. During the validation process, their data were used for validation. There needs to be more external data to verify scoring accuracy and comparative research on predicting the three types of scoring. Therefore, the purpose of the present study was to validate and compare the three scoring systems to determine the most accurate scoring system and provide a reference for clinical selection.

## Methods

The Hospital Ethics Committee approved the study. The STROBE guidelines informed the reporting of this study. [8] The International Classification of Diseases (ICD-10) code for patellar dislocation was used to identify subjects with patellar dislocation. Then, each patient’s inpatient identification number (ID) was obtained from the hospital’s electronic health database. The health records linked to the subjects were obtained as electronic files in the health database. The research was conducted on health records and accessible imaging data. The study population consisted of patients registered in our hospital’s Orthopedic Sports Medicine Center from January 1, 2019, to January 31, 2023. Indicators were measured based on three scoring systems. Patients’ imaging data, including X-ray, CT, and MRI, were reviewed. All radiographic images obtained from the picture archiving and communication system (PACS) were electronic, along with the electronic health records obtained from the hospital information system (HIS). The software used for measurements was Radiant DICOM Viewer (version 5.5.1). All measurement data were measured by one experienced senior orthopedic doctor blindly and randomly simultaneously. The average value calculated by two orthopedic doctors was the final data. If there were abnormal values, another independent, experienced senior orthopedic doctor remeasured them. The inter-observer and intra-observer reliability were measured by the intraclass correlation values (ICC).

**Fig. 1:**
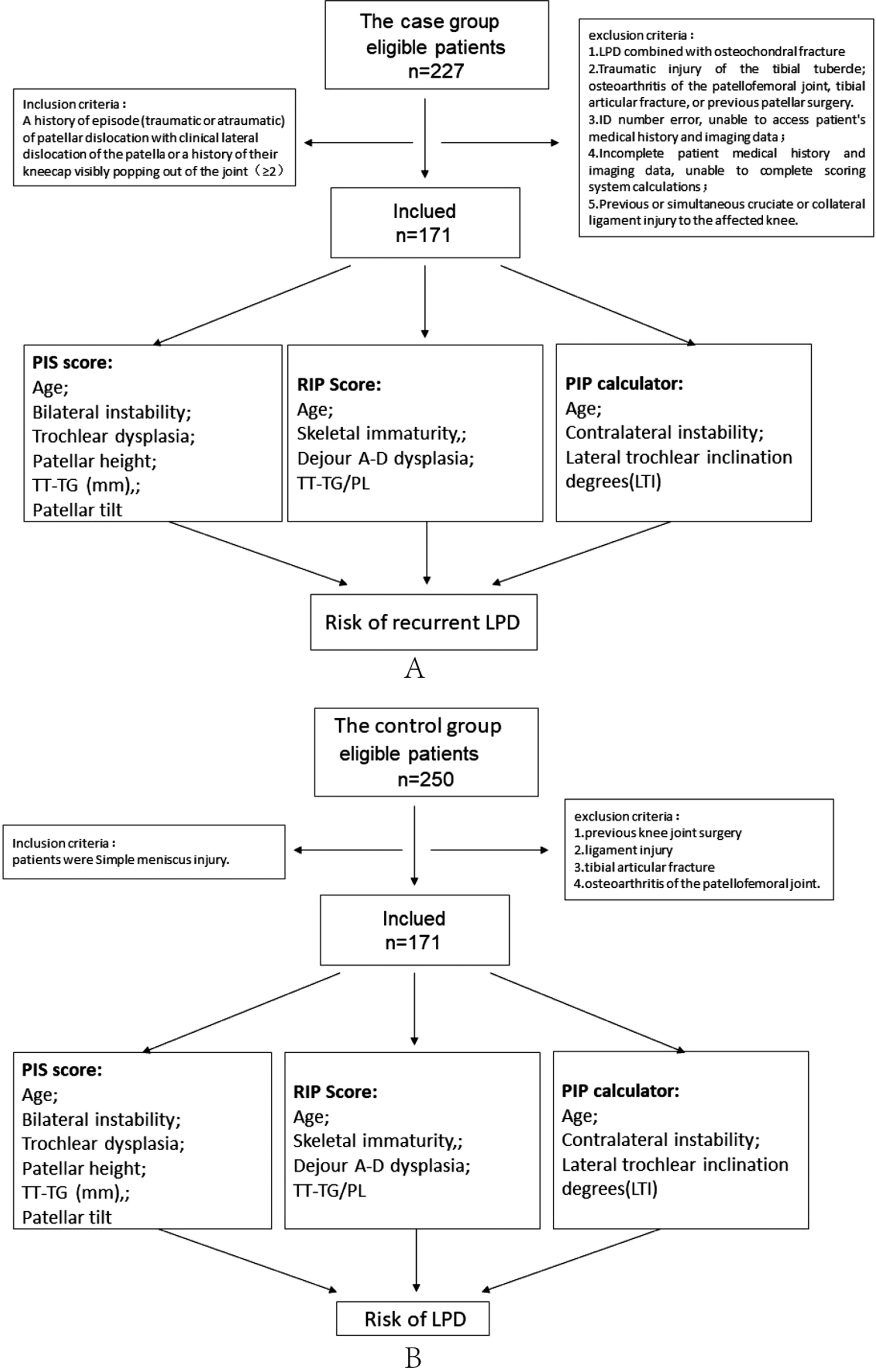
The flow chart of the research

The case group inclusion criteria are patients with recurrent patellar dislocation (patients with objective patellar instability, consisting of at least two patellofemoral dislocations). Exclusion criteria were as follows: LPD combined with osteochondral fracture; traumatic injury of the tibial tubercle; osteoarthritis of the patellofemoral joint, tibial articular fracture, or previous patellar surgery; ID number error, unable to access patient’s medical history and imaging data; incomplete patient medical history and imaging data, unable to complete scoring system calculations; previous or simultaneous cruciate or collateral ligament injury to the affected knee. The patients in the control group had meniscus injuries and needed to be hospitalized for arthroscopic surgery. The inclusion criteria were meniscus injury. Exclusion criteria were as follows: previous knee joint surgery, ligament injury, tibial articular fracture, and osteoarthritis of the patellofemoral joint. There were 227 eligible patients in total in the case group, and due to the exclusion criteria, 56 patients were excluded. The case group consisted of 171 patients (210 knees, age: 20.9 ± 7.08 years old, female/male: 126/45). They were all hospitalized patients with RLPD. There were 250 eligible patients in total in the control group, and due to the exclusion criteria, 79 patients were excluded. The control group consisted of 171 patients (190 knees, mean age: 19.65 ± 8.51 years old, female/male: 121/50). The detailed screening process is shown in Fig. 1.

The patellar instability severity score, the recurrent instability of the patella score, and the patellar instability probability calculator-specific scoring rules and formulas are shown in Table 1. In PIS and RIP, high-risk is defined as RLPD patients, while in PIP, the risk of dislocation ≥ 60% is defined as RLPD patients. Compare the predicted RLPD by the scoring system with the actual situation to determine the accuracy of the prediction.

**Table 1:** Scoring system evaluation rules and formulas

PIS		RIP		PIP
Risk factors	Point	Risk factors	Point	Risk factors
Age(years)		Age(years)		Age(years)
>16	0	≥ 25	0	Contralateral instability
≤ 16	1	< 25	2	LTI
Bilateral instability		Skeletal immaturity		
No	0	No	0	
Yes	1	Yes	1	
Trochlear dysplasia		Dejour A-D dysplasia		
None	0	None	0	
Mild	1	Dejour A-D	1	
Serve	2	TT-TG/PL		
Patellar height(ISI)		<0.5	0	
≤ 1.2	0	≥ 0.5	1	
> 1.2	1			
TT-TG(mm)				
< 16	0			
≥ 16	1			
Patellar tilt (°)				
≤ 20	0			
> 20	1			
RLPD low risk = 0–3 RLPD high risk = 4–7		RLPD low risk = 0–1 RLPD moderate risk = 2–3 RLPD high risk = 4–7		PIP = 1/1 + EXP(-PIOR). PIOR = 3.5 + Age (years)× (-0.1) + contralateral instability×0.7 + LTI× (-0.1)

RLPD: Recurrent lateral patellar dislocation; TT–TG: tibial tuberosity–trochlear groove; ISI: Insall‒Salvati index; PL: Patellar length; LTI: lateral trochlear inclination degrees; PIOR: Patellar instability odds ratio; PIP: Patellar instability probability; EXP: Exponential

### Statistical methods

All analyses were performed using IBM SPSS software (version 25.0, SPSS Inc., Armonk, NY). P < 0.05 was defined as statistically significant. In descriptive analysis, continuous variables are presented as the mean and standard deviation (SD), and discrete variables are presented as frequencies with percentages. The independent-sample t-test and chi-squared test were used to analyze the differences between the case and control groups. Receiver operating characteristic (ROC) curves and the area under the ROC curve (AUC) were measured to assess the diagnostic accuracy of different scoring systems. 0.5 < AUC ≤ 1 indicated predictive ability, and the higher the value, the stronger the predictive ability. AUC ≤ 0.5 indicated no predictive ability. The intraclass correlation coefficient (ICC) and Bland‒Altman plots were used to evaluate the reliability of the measurement data. An ICC value higher than 0.75 indicated good reliability. [9] According to the Bland-Altman plot, all measurement data were within the 95% confidence interval range, indicating a good level of consistency in the data.

## Result

The mean age of the case group was 20.9 ± 7.08 years. There were 45 male (26.3%) and 126 female (73.7%) patients in the case group. Thirty-nine of these patients had bilateral patellar dislocations. Fifty-five cases (32.3%) of trochlear dysplasia were included in the case group. The average age of the control group was 19.65 ± 8.51 years. There were 50 males (29.2%) and 121 females (70.8%), and 29 cases (17%) of trochlear dysplasia in the case group. The two groups had no significant difference in age, sex ratio, or skeletal immaturity (P < 0.05). In the case group, 89 patients (52%) had patella alta (ISI > 1.2), 127 patients (74.3%) had TT-TG ≥ 16 mm, 152 patients (88.9%) had TT-TG/PL ≥ 0.5, and 161 patients (74.3%) had a patellar inclination angle > 20°, with an average LTI of 20.62 ± 4.42°. In the control group, 33 patients (19.3%) had patella alta (ISI > 1.2), 52 patients (30.4%) had TT-TG ≥ 16 mm, 4 patients (2.3%) had TT-TG/PL ≥ 0.5, and 56 patients (32.7%) had a patellar inclination angle > 20°. The average LTI value was 24.73 ± 4.16°, and there was a statistically significant difference between the two groups in patella alta, TT-TG, TT-TG/PL, patellar inclination angle, and LTI (P < 0.05). The detailed descriptive data included in the study are shown in Table 2.

**Table 2:** Characteristics of the patients

	Case group (171)	Control group(171)	P
Age-Y(Mean ± SD)	10–40 (20.9 ± 7.08)	10–47 (19.65 ± 8.51)	0.367
≤ 18	112(65.5%)	100(58.5%)	0.187
>18	59(34.5%)	71(41.5%)	
**Gender**			
Female	126(73.7%)	121(70.8%)	0.546
Male	45(26.3%)	50(29.2%)	
**Bilateral instability**			
No	132 (77.2%)	171 (100%)	
Yes	39 (22.8%)	0 (0%)	
**Trochlear Dysplasia (Dejour)**			
None	116(67.8%)	142(83%)	
A	35(20.5%)	29(17%)	
B-D	20(11.7%)	0(0%)	
**Skeletal immaturity**			
No	65(38%)	58(33.9%)	0.43
Yes	106(62%)	113(66.1%)	
**Patellar height(ISI)**			
≤ 1.2	82(48%)	138(80.7%)	<0.001
> 1.2	89(52%)	33(19.3%)	
**TT-TG/PL**			
<0.5	19(11.1%)	167(97.7%)	<0.001
≥ 0.5	152(88.9%)	4(2.3%)	
Contralateral instability	39(22.8%)	0(0%)	
LTI	20.62 ± 4.42	24.73 ± 4.16	<0.001
**Patellar tilt (°)**			
≤ 20	10(5.2%)	115(67.3%)	<0.001
> 20	161(94.2%)	56(32.7%)	
**TT-TG(mm)**			
< 16	44(25.7%)	119(69.6%)	<0.001
≥ 16	127(74.3%)	52(30.4%)	

In the case group of 171 patients, the PIS was validated in 143 patients with recurrent lateral patellar dislocations. In comparison, in the control group of 171 patients, the PIS was validated in 160 patients who would not experience dislocations. Therefore, the sensitivity of the PIS was 83.6%. The specificity of the PIS was 93.6%. The score’s positive and negative predictive values were 92.9% and 85.1%, respectively (Table 3).

**Table 3:** Diagnostic validity of the PIS for RLPD

		Real situation	
		Positive	Negative
Patellar instability severity (PIS) Score	Positive	143	11
	Negative	28	160

The score’s sensitivity, specificity, positive predictive value, and negative predictive value were 83.6%, 93.6%, 92.9%, and 85.1%, respectively.

In the case group, RIP was validated in 96 patients with recurrent lateral patellar dislocations, while in the control group, PIS was validated in 117 patients who did not experience dislocations. Therefore, the sensitivity of the PIS was 56.1%. The specificity of the PIS was 68.4%. The score’s positive and negative predictive values were 64% and 60.9%, respectively (Table 4).

**Table 4:** Diagnostic validity of the RIP for RLPD

		Real situation	
		Positive	Negative
Recurrent Instability of the Patella (RIP) Score	Positive	96	54
	Negative	75	117

The test’s sensitivity, specificity, positive predictive value, and negative predictive value were 56.1%, 68.4%, 64%, and 60.9%, respectively.

In the case group, PIP was validated in 83 patients with recurrent lateral patellar dislocations, while in the control group, PIS was validated in 50 patients who did not experience dislocations. Therefore, the sensitivity of the PIS was 48.5%. The specificity of the PIS was 29.2%. The score’s positive and negative predictive values were 68% and 36.2%, respectively (Table 5).

**Table 5:** Diagnostic validity of the PIP for RLPD.

		Real situation	
		Positive	Negative
Patellar instability probability(PIP) calculator	Positive	83	121
	Negative	88	50

The sensitivity, specificity, positive predictive value, and negative predictive value of the test was 48.5%, 29.2%, 68%, and 36.2%, respectively.

The intraclass correlation coefficient (ICC) for patellar height (ISI) was strongly consistent (0.922; 95% CI, 0.862–0.956; p < 0.001). The ICC for patellar tilt (°) was strongly consistent (0.941; 95% CI, 0.897–0.966; p < 0.001). The ICC for TT-TG was strongly consistent (0.853; 95% CI, 0.742–0.917; p < 0.001). The ICC for LTI was strongly consistent (0.899; 95% CI, 0.842–0.943; p < 0.001). The ICC for TT-TG/PL was strongly consistent (0.947; 95% CI, 0.907–0.970; p < 0.001). The ICC for trochlear dysplasia was strongly consistent (0.925; 95% CI, 0.869–0.958; p < 0.001). The details are shown in Table 6.

**Table 6:** Results of Intraclass Reliability Calculations (ICC)

Parameter	ICC	95%CI	*P*
Patellar height(ISI)	0.922	0.862–0.956	< 0.001
Patellar tilt (°)	0.941	0.897–0.966	< 0.001
TT-TG	0.853	0.742–0.917	< 0.001
TT-TG/PL	0.947	0.907–0.970	< 0.001
LTI	0.899	0.842–0.943	< 0.001
Trochlear dysplasia (Dejour)	0.925	0.869–0.958	< 0.001
Skeletal immaturity	0.84	0.717 − 0.090	< 0.001

**Fig. 2:**
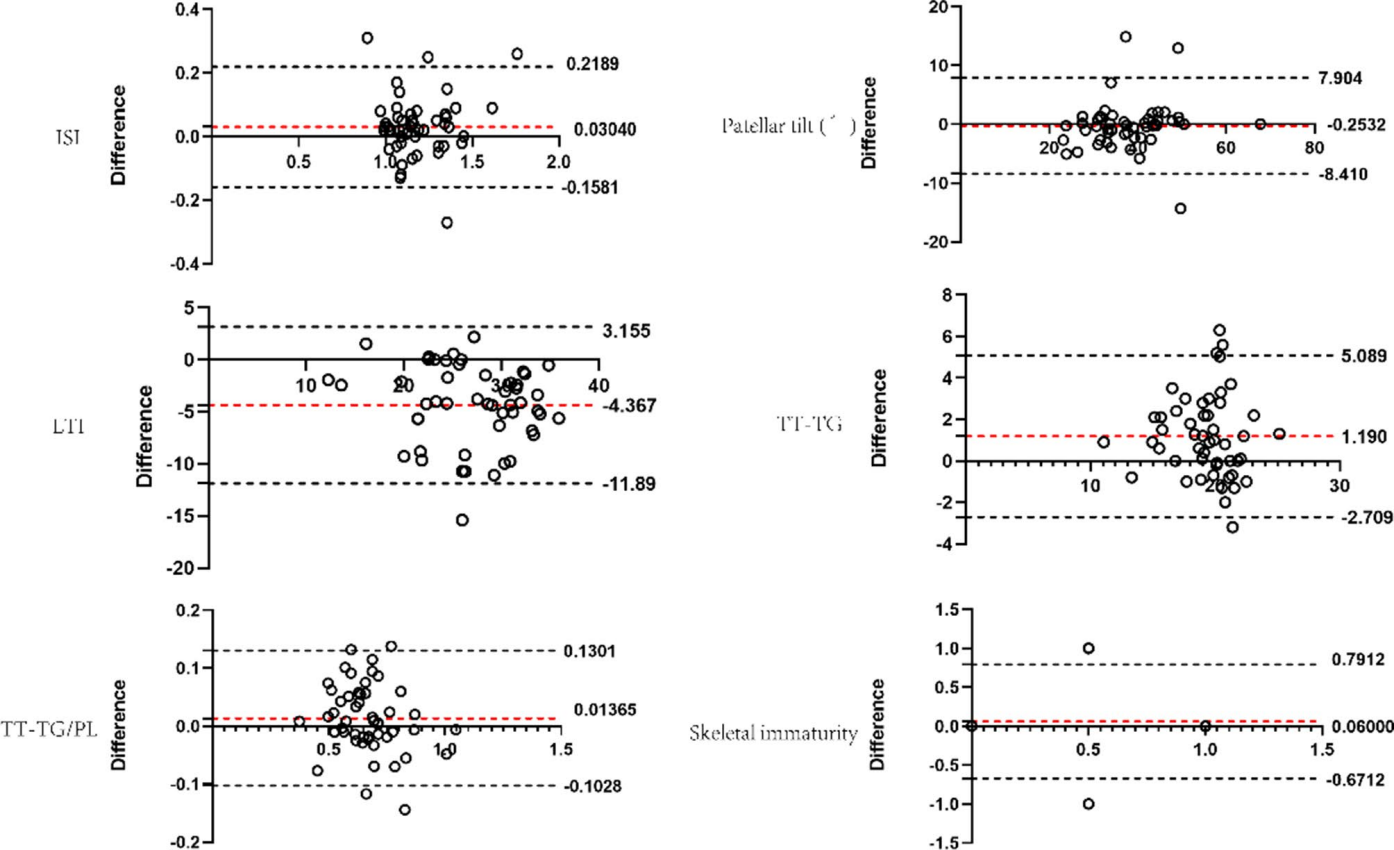
Bland-Altman analysis of the consistency of various measurement indicators

Bland‒Altman analyzed the consistency of Patellar height(ISI)、Patellar tilt (°)、TT-TG、TT-TG/PL、LTI、Skeletal immaturity. The results demonstrate excellent measurement consistency. The details are shown in Fig. 2.

**Fig. 3:**
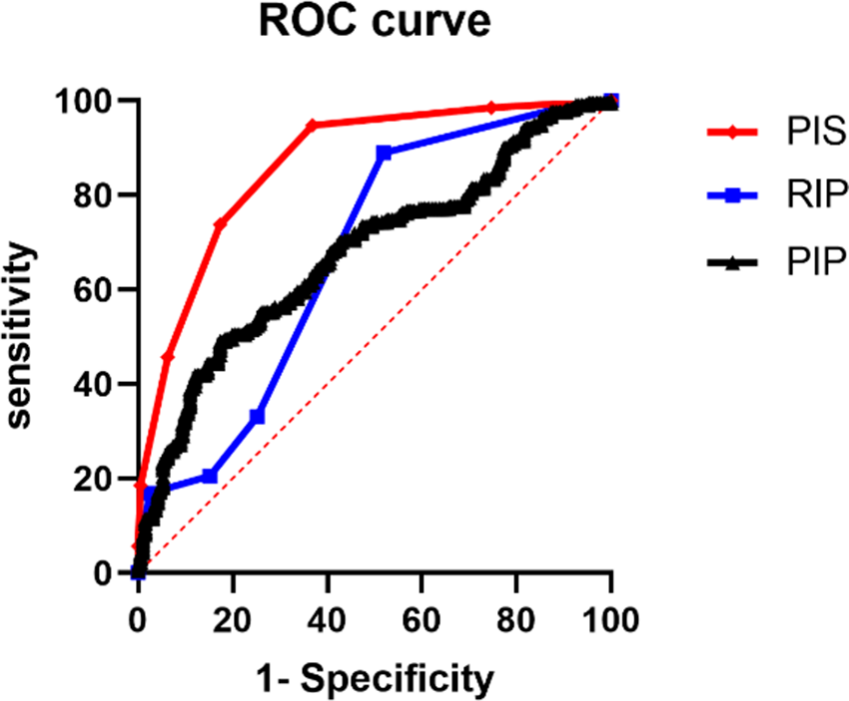
ROC curve showing the AUCs of the PIS, RIP, PIP

For the patellar instability severity (PIS) score, the AUC was 0.866. The sensitivity and specificity were 83.6% and 93.6%, respectively. For the Recurrent Instability of the Patella (RIP) Score, the AUC was 0.673. The sensitivity and specificity were 56.1% and 68.4%, respectively. For the patellar instability probability calculator, the AUC was 0.678. The sensitivity and specificity were 48.5% and 29.2%, respectively. The above results indicated that the diagnostic accuracy of the PIS was better than that of the PIP and RIP. The details of the AUC are shown in Table 7; Fig. 3.

**Table 7:** Diagnostic performance of the scoring system

Scoring system	AUC	95% confidence interval	*P*
PIS	0.8666	0.8312–0.9019	< 0.0001
RIP	0.673	0.6195–0.7251	< 0.0001
PIP	0.6786	0.6265–0.7308	< 0.0001

## Discussion

In this study, the validity of the patellar instability severity (PIS) score, the recurrent instability of the patella (RIP) score, and the patellar instability probability calculator (PIP) were evaluated. This study is the first to externally validate three models for predicting recurrent patellar dislocation using external data. Compared to RIP and PIP, PIS has a relatively more accurate predictive ability. Validation studies revealed that RIP showed 83.6% sensitivity and 93.6% specificity in predicting recurrent lateral patellar dislocation. The AUC was 0.866 (95% CI 0.8312–0.9019). Therefore, PIS can be recommended as an effective method for predicting recurrent dislocation in patients with recurrent lateral patellar dislocation. PIS is essential in determining the treatment plan for patients with recurrent lateral patellar dislocation.

The main risk factors for recurrent patellar dislocation include age, history of contralateral patellar dislocation, patellar tilt, patellar height, trochlear dysplasia, distance from the tibial tubercle to the trochlear groove (TT-TG), TT-TG/patellar length (PL) ratio, distance from the tibial tubercle to the lateral trochlear ridge (TT-LTR), and distance from the tibial tubercle to the posterior cruciate ligament (TT-PCL), etc. The indicators used in the multiple regression models for predicting the individual risk of recurrent lateral patellar dislocation are not entirely the same.

Patient age is an essential factor for patients with recurrent patellar dislocation. All three scoring systems included age as a risk factor in the calculation. Although the mechanism underlying the association between age and dislocation is not yet clearly defined, younger patients are believed to have an increased risk of recurrent events due to age. [10] In the case group, the average age was 20.0 ± 7.08 years old. Age indicators play an essential role in predicting patients with recurrent patellar dislocation. In PIS, age is stratified into 16 years old and assigned, while in RIP, age is stratified into 25 years old and trusted. According to Christensen et al., the risk of recurrence in patients with primary dislocation under 18 is more than twice that of similar adult patients. [11] Compared to patients over 16.6 years old, patients under 16 have a risk of more than 11 times higher. [5] Therefore, PIS layering is more accurate.

The previous instability of the ipsilateral or contralateral patella increases the risk of recurrence of ipsilateral patellar dislocation by three times compared to patients who have not previously experienced patellar dislocation. [5, 10] Bilateral instability is a significant risk factor for predicting recurrent patellar dislocation. [11] PIS and PIP included bilateral instability as a risk factor in the score, while RIP did not, which may be the reason for decreased RIP prediction ability.

In recent literature, dysplasia of the femoral condyle has been identified as one of the strongest predictors of recurrent instability, with an increased risk of 2.6 to 23.7 times compared to knee joints without developmental abnormalities. [5, 11, 12] It leads to instability by interfering with patellar tracking and alignment throughout the entire range of knee motion. [10] The Dejour classification method is commonly used to classify femoral condylar dysplasia. [13] In PIS, femoral condylar dysplasia is classified as none, mild, and severe, with scores of 0, 1, and 2, respectively. RIP is classified as none and Dejour A-D, with scores of 0 and 1, respectively. PIP was not considered a risk factor. In the study, there were 55 cases (32.2%) of femoral condylar dysplasia in the case group, including 35 patients (20.5%) of Dejour A and 20 points (11.7%) of Dejour B-D. In the control group, there were 29 cases (17%) of Dejour A. The critical role of femoral condylar dysplasia in predicting recurrent patellar dislocation should be included as an essential risk factor in the scoring system. Compared with RIP, the PIS assignment distribution is more reasonable.

Many studies suggest that the patella alta has long been closely related to recurrent lateral patellar dislocation. The risk of dislocation increased by 1.6 to 10.6 times compared to non-patella alta. [11, 12, 14, 15] In the case group, there were 89 cases (52%) of patella alta, while in the control group, there were 33 cases (19.3%) (P ≤ 0.05). Therefore, patella alta is a risk factor for recurrent patellar dislocation. In the three scoring prediction systems, RIP and PIP did not include patella alta as a risk factor in the score, and only PIS had it, which may be the reason for the poor predictive ability of RIP and PIP scores.

There is strong evidence in the literature that increasing the TT-TG distance is a significant risk factor for recurrent lateral patellar dislocation. [16–19] Arendt et al. confirmed that the average TT-TG of primary lateral patellar dislocation was 15.6 mm, and there was no significant difference between skeletal maturity (age range 11–50 years) and immaturity (age range 10–17 years). [20] In our study, there were 127 cases (74.3%) with TT-TG ≥ 16 mm in the case group and only 52 cases (30.4%) in the control group. P ≤ 0.05. PIS and RIP included TT-TG as a risk factor in the scoring system, but TT-TG was not included in PIP, which may be one of the possible reasons for the insufficient predictive ability of PIP.

Ahrend et al. [21] and Balcarek et al. [5]. have shown that patellar tilt (≥ 20°) is a risk factor for recurrent patellar dislocation. The risk of dislocation increased by 1.93 times compared to patellar tilt (< 20°). In our study, consistent with literature reports, patellar tilt ≥ 20° was significantly higher in the case group than in the control group. 161 cases (94.2%) and 56 patients (32.7%), respectively, P < 0.05. PIS is used as a risk factor for predicting recurrent patellar dislocation, which is one of the possible factors that PIS has more vital predictive ability than PIP and RIP.

Receiver operating characteristic (ROC) curves and the area under the ROC curve (AUC) were established to assess PIS, RIP, and PIP prediction accuracy. The AUCs were 0.866, 0.673, and 0.678, respectively. Wierer et al. validated the predictive accuracy of PIP and PIS using ROC and AUC when proposing and creating PIP, with ACUs of 0.66 and 0.79, respectively. [7] This difference is because they verify through internal data and lack support from external data. Second, the sample size included in the study was relatively small, with a total of 201 cases included. We had 342 patients, which may also be the reason for the difference in results.

### Limitations

Limitations include the data from a single center and the study’s retrospective design. We only collected indicators from the PIS, RIP, and PIP scoring systems. We did not contain any other risk factors reported in the literature that may be associated with a recurrent lateral patellar dislocation, such as local torsion of the distal femur, lower limb force line deformity, or body mass index. [7, 22, 23] Although our data come from a single center, we are a medical Center in the region with many patients and comprehensive coverage. Therefore, the data are representative. We have validated PIS, RIP, and PIP through a large amount of data, and the results indicate that PIS has a better ability to predict recurrent lateral patellar dislocation than RIP and PIP. Further validation by multicenter independent investigators using external data sets is recommended.

## Conclusions

The study findings indicated that the PIS is more reliable for evaluating recurrent lateral patellar dislocation than the RIP and PIP. RIP and PIP didn’t work, as their AUCs were < 0.70. PIS can accurately predict recurrent lateral patellar dislocation. It is an effective method for predicting recurrent patellar dislocation in outpatient and inpatient patients. It can aid doctors in making treatment decisions.

## Data Availability

The datasets supporting the conclusions of this article are included within the article. Raw data can be requested from the corresponding author.
